# Hydrastis Canadensis in Gonorrhœa

**Published:** 1851-11

**Authors:** D. M. McCann

**Affiliations:** Martinsburgh, Ohio


### RECORD OF MEDICAL SCIENCE.

### SURGERY.

*Hydrastis Canadensis in Gonorrhœa.* By Dr. D. M. McCann, M.D., Martinsburgh, Ohio.—As your excellent Medical Journal has for its object the diffusion of knowledge advantageous to the Medical profession, permit me to call the attention of the profession through its columns to the use of Hydrastis canadensis, (yellow root, orange root,) in gonorrhœa.

I am not aware that any of my brethren have ever used it in this affection, before myself. My experience, however, in the use of it, though not extensive, is yet sufficient to warrant me in soliciting a trial of it by those having more opportunity of testing its curative power than I have. I have used it in several cases in various stages of the disorder, and in *every case* with the most satisfactory results; more especially with males than females.

I was led to its use by noticing its well known sanative properties over inflammations of mucus and epithelial structures, such as aphthæ of the the mouth, &c. The ardor urinæ, and discharge of mucus, had been entirely suspended in every case in from twenty-four to seventy-two hours. In some cases, I used the balsam copaibæ, in others, injections of infusion of the hydrastis alone, but with about the same results, a perfect and *permanent* eradication of the disorder.

I have varied the strength to suit the case in its different stages, but as a general rule I have used about one drachm of the dried root to the pint of infusion—injecting a syringe full three or four times a day.

I hope that some of the profession will give this article a fair trial.—*Ohio Med. and Sur. Journal.*

About the time of our commencing the practice of medicine, ten years ago, our attention was called to the use of the infusion of yellow root as an injection in gonorrhœa, by a skilful and experienced physician of this city, Dr. U. E. Ewing. At his suggestion we employed it ourself, and with such good results, that we have continued, to the present time, to use it in cases in which astringent injections were indicated. A strong infusion of this root containing a small proportion of sulphate of copper in solution, forms one of the best injections for gonorrhœa we have ever used; and for some years after we had our attention called to it by Dr. E. we used it almost exclusively, preferring it to any injection that we had employed. Of late years, however, we have used the solution of chloride of zinc, not because of its superior efficacy, but simply because it is more readily and conveniently prepared. We have tried nearly, if not quite all, the injections we have seen recommended for gonorrhœa, and we have no hesitation in giving the two already mentioned the preference. With the infusion of yellow root alone, or containing sulphate of copper in solution, and the solution of chloride of zinc, we think all the good may be accomplished in the treatment gonorrhœa which can be gained by injections.

The formula of the infusion of yellow root, as directed by Dr. Ewing, which he has employed upwards of twenty years, is as follows:

Pulv. Hydrastis Canadensis, i.
Aquæ Puræ, f℥viij.
M.

Digest for several hours and strain.

To this Dr. E. is in the habit of adding sulph. cupri ʒi. We have directed this injection repeatedly in confirmed cases of gonorrhœa and gleet, and our experience confirms the favorable report of its value, which we received from Dr. Ewing ten years ago.—*Transylvania Med. Jour.*

